# Metabolomic approaches suggest two mechanisms of drought response post‐anthesis in Mediterranean oat (*Avena sativa* L.) cultivars

**DOI:** 10.1111/ppl.70181

**Published:** 2025-03-27

**Authors:** Aiswarya Girija, Francisco J. Canales, Bahareh Sadat Haddadi, Rachel Dye, Fiona Corke, Kevin Williams, Helen Phillips, Manfred Beckmann, Elena Prats, John H. Doonan, Luis A. J. Mur

**Affiliations:** ^1^ Department of Life Sciences, Penglais campus Aberystwyth University UK; ^2^ Institute of Biological, Environmental & Rural Sciences (IBERS) Aberystwyth University; ^3^ Agroforestry and Plant Biochemistry, Proteomics and Systems Biology, Department of Biochemistry and Molecular Biology University of Cordoba Spain; ^4^ The National Plant Phenomics Centre Aberystwyth University UK; ^5^ CSIC‐Institute for Sustainable Agriculture Spain

## Abstract

Oats (*Avena sativa* L) is a temperate cereal and an important healthy cereal cultivated for food and feed. Therefore, understanding drought responses in oats could significantly impact oat production under harsh climatic conditions. In particular, drought during anthesis (flowering) affects grain filling, quality and yield. Here, we characterised metabolite responses of two Mediterranean oat (*Avena sativa* L.) cultivars, Flega and Patones, during drought stress at anthesis. In the more drought‐tolerant Patones, the developing grains from the top (older) and bottom (younger) spikelets of primary panicle were found to be larger in size in response to drought, suggesting accelerated grain development. Flega showed a more rapid transition to flowering and grain development under drought. The metabolomes of source (sheath, flag leaf, rachis) and sink (developing grains) tissues from Patones showed differential accumulation in fatty acids levels, including α‐linolenic acid, sugars and amino acids with drought. Flega showed enhanced energy metabolism in both source and sink tissues. Lower levels of glutathione in source tissues and the accumulation of ophthalmic acid in the grains of Flega were indicators of oxidative stress. Our study revealed two distinct metabolite regulatory patterns in these cultivars during drought at anthesis. In Patones, α‐linolenic acid‐associated processes may accelerate grain‐filling, while in Flega oxidative stress appears to influence traits such as flowering time. Overall, this work provides a first insight into the metabolite regulation in oat's source and sink tissues during anthesis under drought stress.

## 1 INTRODUCTION

Drought is a major abiotic stress in cereals affecting agriculture and global food security. Drought can affect crops at any stage of their development, impacting on morphological, physiological, and molecular processes, which affects nutrient balance, growth and yield (Schmidt et al., 2023). Therefore, to withstand future drought‐prone environments and to improve crop productivity, cultivars with better drought resilience need to be developed (Rispail et al., 2018). Drought tolerance is linked to crop strategies leading to maintain cellular water and osmotic homeostasis (aquaporins, Patel and Mishra, 2021), protect membranes (dehydrins, Yu et al., 2018), and trigger metabolic shifts leading to the accumulation of osmolytes or increased cuticular waxiness (Patwari et al., 2019). In the other hand, drought avoidance mechanisms aim to minimise water loss in plants; they can involve developmental changes such as deeper root penetration into the soil, stomatal closing or leaf rolling. Another mechanism through which plants deal with drought is to accelerate the developmental cycle and reproduce to form seeds and escape the stress (Kooyers, 2015; Bandurska, 2022). To enhance crop performance, it is crucial to understand these mechanisms and how plants respond and adapt to drought.

In cereals, anthesis and grain‐filling are the most sensitive and important developmental stages determining the yield potential. Hence, drought occurring at anthesis or grain‐filling stage has severe effects on plant growth, crop yield and quality. The grain‐filling process is controlled by complex physiological, metabolic and cellular events that may be directly and/or indirectly influenced by abiotic and biotic factors (Ma et al., 2023). Grain‐filling is influenced by grain number and size and final grain size is correlated to grain‐filling capacity (McCabe & Burke, 2022). The total grain weight in wheat and barley is determined by the grain‐filling rate, whereas the seed‐filling duration is more important in rice and both duration and rate are crucial for maize's grain weight (Haverroth et al., 2021; Kennedy et al., 2018). These different processes are dependent on source and/or sink availability. Sink strength during the reproductive stage arises from the ability of grains to accumulate dry matter, which is defined by the number and weight of grains (Slafer et al., 2023). The ability of crops to accumulate photo‐assimilates from sources such as stem and leaf to sink is also relevant (Du et al., 2024; Rosado‐Souza et al., 2023). In wheat, yield is found to be sink‐limited during grain‐filling and partially relies on the remobilization of resources stored in stem, spike and leaf (Rosati & Benincasa, 2023). Drought stress at post‐anthesis stage alters photosynthetic efficiency and the re‐mobilization of assimilates from source to developing grains (Shirdelmoghanloo et al., 2022). Reduced water levels during grain‐filling lead to a diminished photosynthetic activity, which affects the mobilisation of nutrients from source to developing grains, leading to poor grain development and yield (Yan et al., 2021). Such photoassimilate partitioning is determined by many factors, including genotype, environment, irrigation, fertilisation, plant density, and photosynthetic ability (Tovignan et al., 2020). By increasing our understanding of how photoassimilate partitioning occurs, it may be possible to improve yield potential in crops growing under drought‐prone climates.

Oat (*Avena sativa* L.) is an economically important cereal crop of Mediterranean origin. Oat grains are rich in protein (avenins), lipids (3–18% fatty acids such as oleic, linoleic and palmitic acid), amino acids (lysine), antioxidants (e.g. avenanthramides), dietary fibres such as β‐glucan, and phenolics (tocols and saponins) (Allwood et al., 2021). With its nutritional and health benefits, oats have gained attention in the food, pharmaceutical and cosmetic industries (Allwood et al., 2021). Compared to other cereals, oats are well‐adapted to marginal environments and can grow in marginal soils with low nutrient levels (Canales et al., 2021; Kutasy et al., 2021). Being a temperate cereal, oat is sensitive to hot, dry environmental conditions and is more susceptible to grain abortion and poor grain development under drought (Kutasy et al., 2021). This makes oat vulnerable to water‐limiting conditions, which limit its yield (Konieczna et al., 2023). Therefore, understanding oat response to drought is important to alleviate the impact of drought stress on oat production and quality.

In this study, we applied untargeted metabolomic approaches to characterise the effect of drought stress at anthesis in two commercial Mediterranean oat cultivars (cv): ‘Flega’ and ‘Patones’. At the seedling stage and under field conditions, Flega is known to be drought‐susceptible (Canales et al., 2021) and Patones to be drought‐tolerant (Sánchez‐Martín et al., 2018; Sánchez‐Martín et al., 2015; Sánchez‐Martín et al., 2014). We hypothesised that the differential responses of these two genotypes to drought would be reflected in discrete metabolic shifts in source tissues (sheath, flag leaf and rachis) and developing grains (sink) of the primary panicle. The identified metabolic pathways could be further explored to improve oat grain quality and yield under drought.

## 2 MATERIALS AND METHODS

### 2.1 Plant Material

The experiment was conducted at National Plant Phenomics Centre (NPPC), Institute of Biological, Environmental & Rural Science (IBERS) at Aberystwyth University, Wales, UK (52.43 N, 4.01 W) from January to March 2022. The two oats (*Avena sativa*) cultivars, Flega and Patones, used for the study are Mediterranean commercial cultivars. Seeds of Flega and Patones were provided by the CSIC‐Institute for Sustainable Agriculture, Spain. Flega was developed by the Cereal Institute (Thermi‐Thessaloniki, Greece) and Patones was developed by the ‘Instituto Madrileño de Investigación y Desarrollo Rural, Agrario y Alimentario’ (IMIDRA, Madrid, Spain). Compared to Flega, Patones exhibits good adaptation to Mediterranean agroclimatic conditions (Sánchez‐Martín et al., 2014; Canales et al., 2021). Details of the genetic relationships between these cultivars have been previously reported and showed that they are not closely related (Montilla‐Bascón et al., 2013). The seeds of two cultivars were sown in 3.5‐L plastic pots in LemnaTec platform that contained experimental soil mix, Levington F2 a peat‐based compost (pH 5.3–5.8; N 144: P 73: K 239). The plants were grown at ambient CO_2_, light intensity of 450 μmol m^−2^ s^−1^ photosynthetic photon flux density (PPFD) under natural light supplemented with artificial light for 14‐h photoperiod. The average temperature during the experiment was 20°C.

### 2.2 Drought treatment in adult plants at anthesis

A total of 32 plants (8 plants per treatment with one plant per pot x 2 treatments x 2 genotypes) were grown to the developmental growth stage (GS) GS55 (panicle half emerged (Zadoks et al., 1974)). Once the plants reached GS55 (half panicle emerged), eight pots per genotype were set aside to undergo progressive drought by withholding water (the other 8 were irrigated as usual and consider as control). 7 days after the start of drought, the soil relative water content reached (SWC) 25% and plants flowered and entered anthesis (GS61). The targeted SWC (25%) was maintained by the automatic weighing and watering station that forms part of the plant conveyor belt system within the NPPC (https://www.plant-phenomics.ac.uk/index.php/resources/lemnatec-system/) for 15 days throughout anthesis (GS61) to grain‐filling (GS75). After 15 days, the primary panicles from control and drought groups were harvested for metabolite analysis. On the day of sampling, plants were at growth stage GS75 (grains at milky stage). Upon harvest, the number of whorls and number of spikelets were recorded from the primary panicle. Sheath (S), flag leaf (Fl), rachis (R) and spikelets were sampled from the primary panicle for metabolite extraction with a sample size of *n* = 8 replicates for each group for both cultivars (Figure S1). Harvested tissues were flash‐frozen in liquid N2 and stored at −80°C. After sampling, the plants were maintained at the same soil water capacity and, upon maturity, shoot biomass, number of tillers, stem height, number of panicles, total panicle weight, number of whorls and number of spikelets were recorded.

### 2.3 Stomatal observation

Leaf water conductance of flag leaves was recorded from the mid of the adaxial surface of leaf laminae using an AP4 cycling porometer (Delta‐T Devices Ltd). Fv/Fm was also measured throughout the drought experiment using a Handy PEA + system (Hansatech Instruments Ltd). Both stomatal conductance and F_v_/F_m_ were measured from the flag leaf (FL) of the primary tiller of control (n = 8) and drought‐treated plants (n = 8). All the measurements were carried out in the flag leaf of the primary tiller 6 h after the onset of the light period.

### 2.4 Metabolite profiling of sheath, flag leaf, rachis and developing grains

Metabolites from milled samples from sheath, flag leaf, rachis and developing grains of Flega and Patones from control and drought groups were extracted. About (20 ± 1 mg) samples were extracted in 500 μL of extraction buffer with chloroform/methanol/water (1:2.5:1, v/v/v) followed by incubation at 4°C for 15 min. Following microcentrifugation (21,000 x *g*, 4°C), the supernatant with both polar and non‐polar fractions was collected and transferred for metabolite profiling. An aliquot of 100 μL of the supernatant was transferred to an HPLC glass vial with a 0.2 mL micro insert (Kattupalli et al., 2021; Allwood et al., 2006). Untargeted metabolite profiling was undertaken by Flow infusion electrospray high‐resolution mass spectrometry (FIE‐HRMS) based on a Q Exactive plus Hybrid Quadrupole Orbitrap Mass analyser with an Acella ultra‐high‐performance liquid chromatography (UHPLC) system (Thermo Fisher Scientific©). The samples were injected in a randomized order into the capillary column. Mass‐ions (*m/z*) were acquired in both positive and negative ionisation modes and data were normalised to the total ion count.

### 2.5 Metabolite annotation and statistical analysis

Statistical analyses were performed on log_10_‐transformed values and normalized with Pareto scaling. Multivariate analysis and visualization were performed using the online tool Metaboanalyst 5.0 (https://www.metaboanalyst.ca/MetaboAnalyst/). The significant *m/z* features were identified using one‐way analysis of variance (ANOVA) and post‐hoc test using Tukey's Honestly Significant Difference (Tukey's HSD) and Fisher Least‐Significant Difference (LSD) test (*p <* 0.05). Principal component analysis (PCA) and hierarchical cluster analyses (HCA) were done to visualize the metabolite patterns across samples with the online R‐based platform Metaboanalyst 5.0 (Pang et al., 2022). The differentially regulated metabolites between control and drought groups in two cultivars were determined by fold change (FC) thresholds >2.

The significant metabolites were identified based on accurate masses (mass tolerance = 5 ppm resolution) and functional enrichment was done using the *Oryza sativa* japonica database in the Kyoto Encyclopaedia of Genes and Genomes (KEGG)(http://www.genome.jp/kegg/) and also with the Human metabolite (HMDB), PubChem, and ChEBI databases. Metabolite identifications were done considering the following possible adducts: [M^+^]^+^, [M + H]^+^, [M + NH_4_]^+^, [M + Na]^+^, [M + K]^+^, [M‐NH_2_ + H]^+^, [M‐CO_2_H + H]^+^, [M‐H_2_O + H]^+^; [M−]^−^, [M − H]^−^, [M + Na − 2H]^−^, [M + Cl]^−^, [M + K − 2H]^−^.Correlations between multiple adducts of the suspected metabolites were used in the identification process and heatmaps were used to map the metabolites with Pearson r distances (Table S1).

### 2.6 Data Analysis for Morphological, Physiological and Yield Traits

The overall effect of drought on the morphological and physiological traits was assessed by mixed‐model analyses of variance (ANOVA) with cultivars and treatment as the main effects. The results were expressed as mean values and error bars represent standard deviation (SD). A one‐way analysis of variance (ANOVA) for water data was used to investigate the effect of the cultivar on drought treatment. All statistical analyses were completed using R statistical Software (https://www.r-project.org/), JMP software (https://www.jmp.com/en_gb/home.html) and figures were generated using Graphpad Prism 8.0.0 (https://www.graphpad.com/features).

## 3 RESULTS

### 3.1 Effect of drought on morphological and physiological traits

**FIGURE 1:**
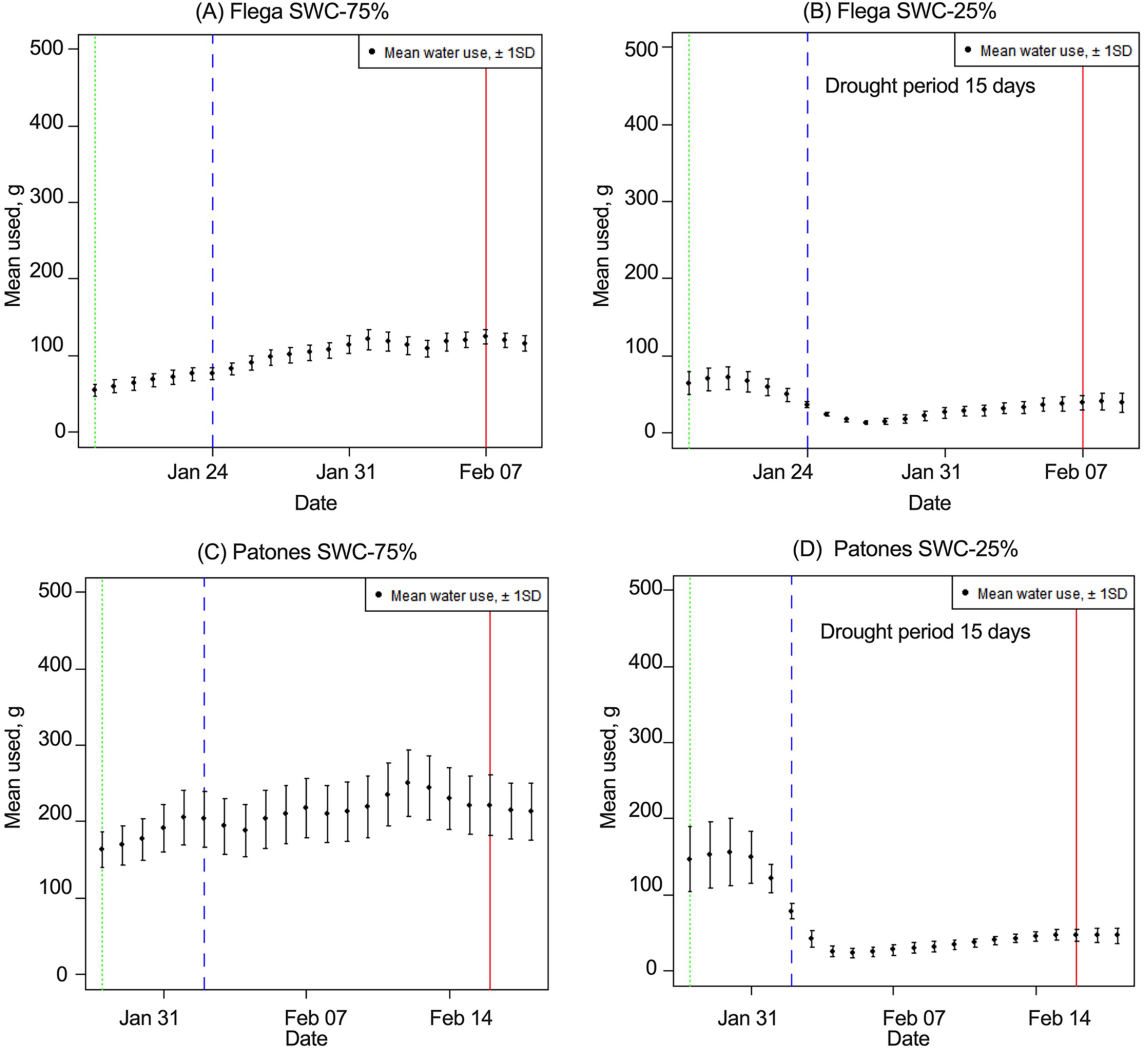
Daily cumulative water used (g) over the drought period for 15 days from GS61 (A) Flega grown under control conditions, SWC 75% (B) Flega grown under drought conditions, SWC 25% (C) Patones grown under control conditions, SWC 75% and (D) Patones grown under drought conditions, SWC‐25%. The black dotted line indicates the day at which SWC_25% has reached and red solid line shows the day when the primary panicle was sampled. Data is represented as daily mean ± SD, *n* = 8.

**FIGURE 2:**
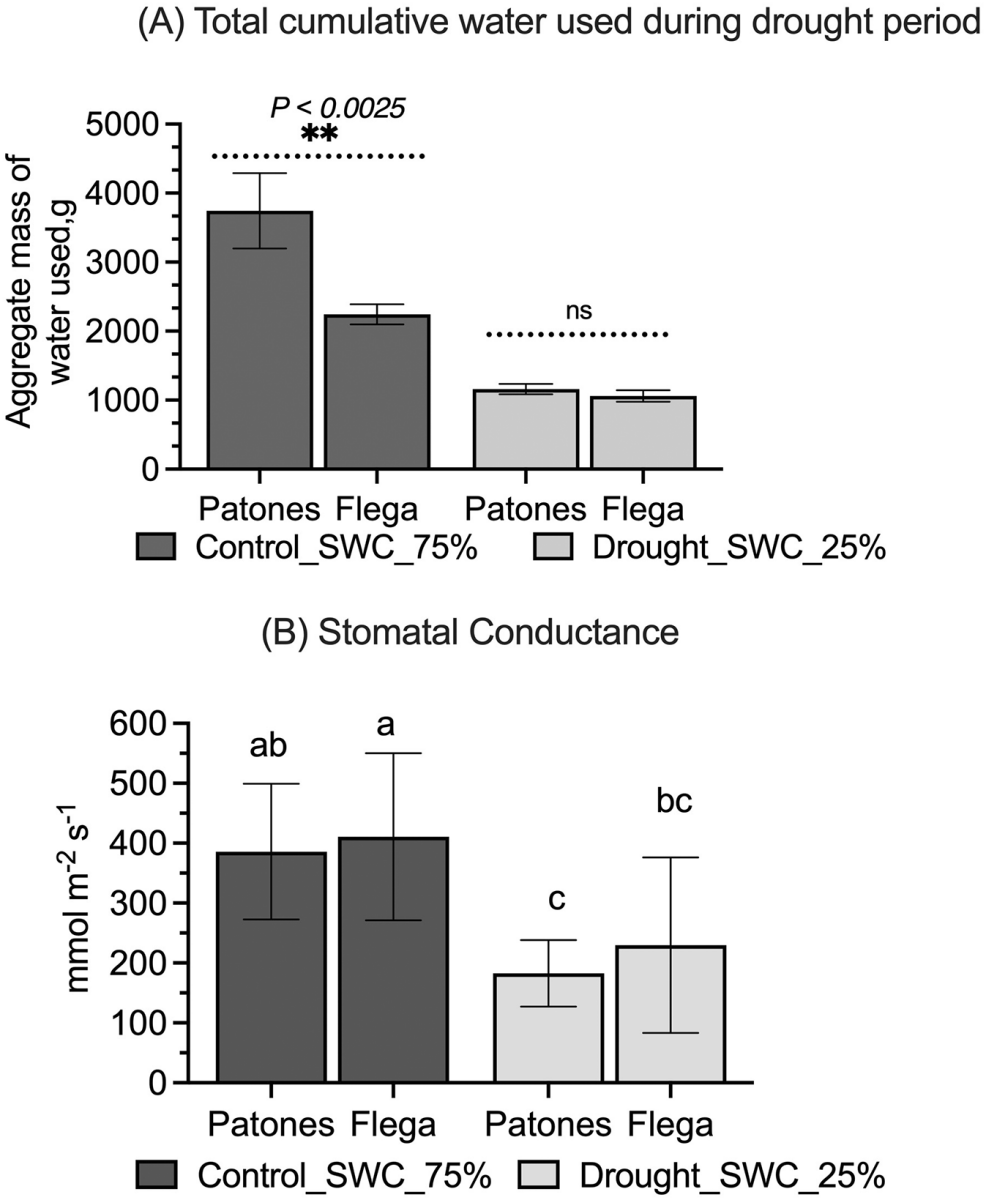
(A) Total mean cumulative water used (in g) over the drought treatment for 15 days from GS61 in Flega and Patones grown under control conditions (SWC‐75%) and drought conditions (SWC_25%). Compared to Patones, Flega showed less water usage under control. Data is represented as mean ± SD, n = 8 and statistical significance using one‐way ANOVA, *p* < *0.05* is shown as ‘*’ and non‐significance as ‘ns’. (B) Stomatal conductance of flag leaf from the primary panicle at the end of drought treatment in Flega and Patones under control (SWC_75%) and drought (SWC_25%) conditions. The data is represented as mean ± SD, n = 8 and significant differences using ANOVA Tukey HSD test, *p* < *0.05* are identified by different letters.

Compared to well‐watered conditions, total plant water usage in Flega and Patones was signifcanty (*p* < *0.05*) reduced during the drought period from GS61 (Figure 1). The black dotted line in Figure 1 indicates the day at which SWC 25% was reached and red solid line shows the day when the primary panicle was sampled. The data were aggregated for all eight replicates with each treatment for both cultivars to derive cultivar water use data. Both cultivars showed reduced total cumulative water use under drought. Interestingly, we found that Flega was distinct from Patones and showed a significant (*p* < *0.05*) reduction in total water usage under both control and drought conditions (Figure 2A). Stomatal conductance from the flag leaf of the primary panicle was equally reduced in both cultivars under drought (Figure 2B). We also measured F_v_/F_m_, and this did not significantly change with the drought treatment in both cultivars (data not shown).

**FIGURE 3:**
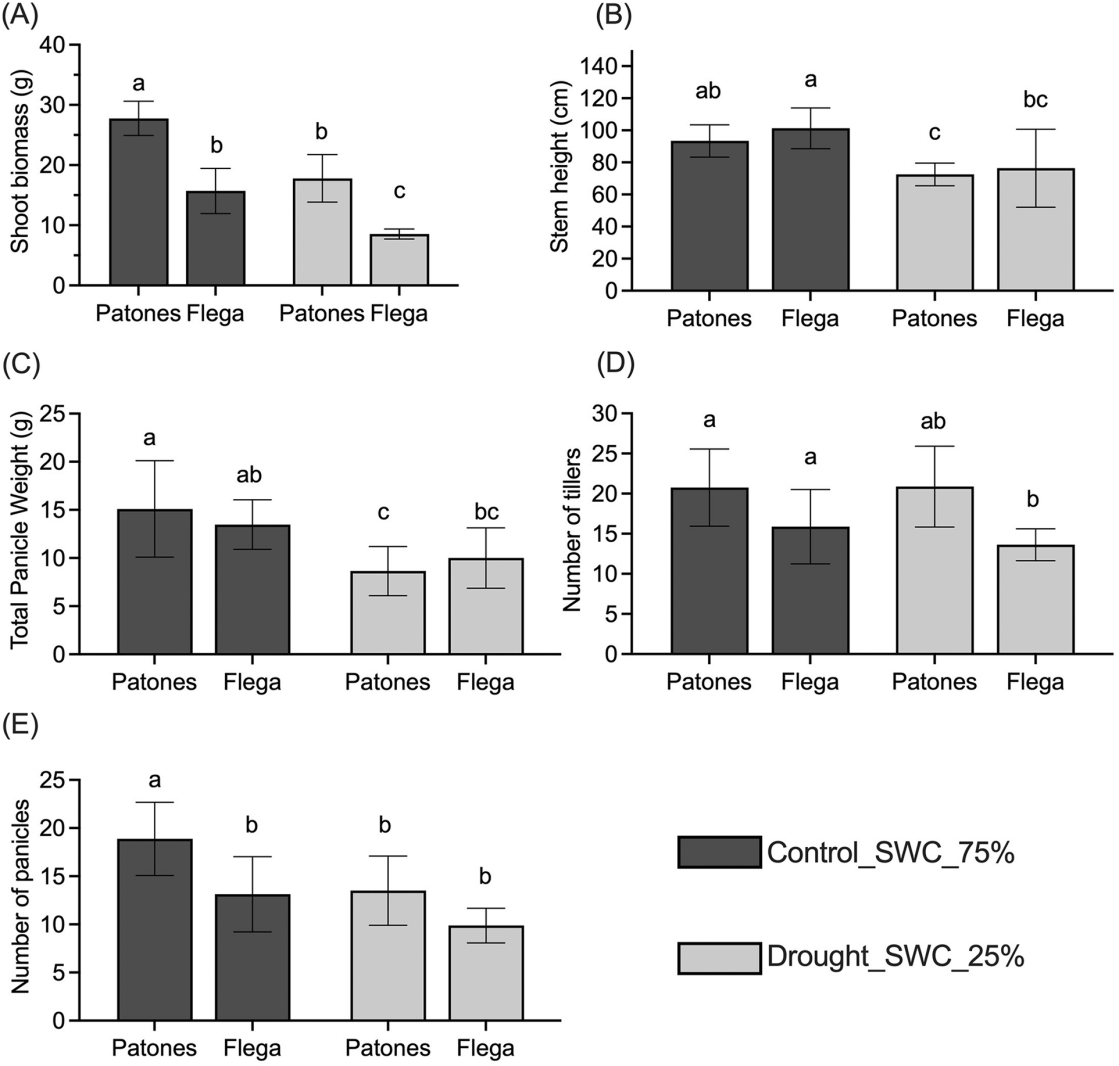
(A) Shoot biomass (B) Stem height (C) Total panicle weights (D) Number of tiller and (E) Number of panicles in Flega and Patones under control and drought conditions. The data is represented as mean ± SD, n = 8 and significant differences using ANOVA Tukey HSD test, *p* < *0.05* are identified by different letters.

Significant differences were observed in shoot biomass, stem height, number of tillers, number of panicles, total panicle weight of both cultivars under drought (Figure 3). Both cultivars showed reduced shoot biomass under drought; however, compared to Flega, Patones showed a significantly greater reduction in shoot biomass, stem height and total panicle weight under drought (Figure 3A, B,C). Compared to control plants, Flega showed a reduced number of tillers under drought but no significant change in the number of panicles (Figure 3 **D, E**). Under both conditions, Flega showed faster growth, which allowed the cultivar to reach flowering stage a week earlier than Patones. This could allow it to complete its life cycle quickly to escape stresses such as drought.

### 3.2 Evaluation of developing grain phenotype under drought

The number of spikelets in each floret is influenced by factors such as grain number and grain‐filling rate (Haverroth et al., 2021). Thus, each spikelet on a given panicle can represent a developmental series that reflects grain development. In oats, grain maturation begins at the panicle tip and proceeds towards the base. In a spikelet, the florets develop acropetally, and floret numbers can vary considerably (typically 1 to 4) in each spikelet.

**FIGURE 4:**
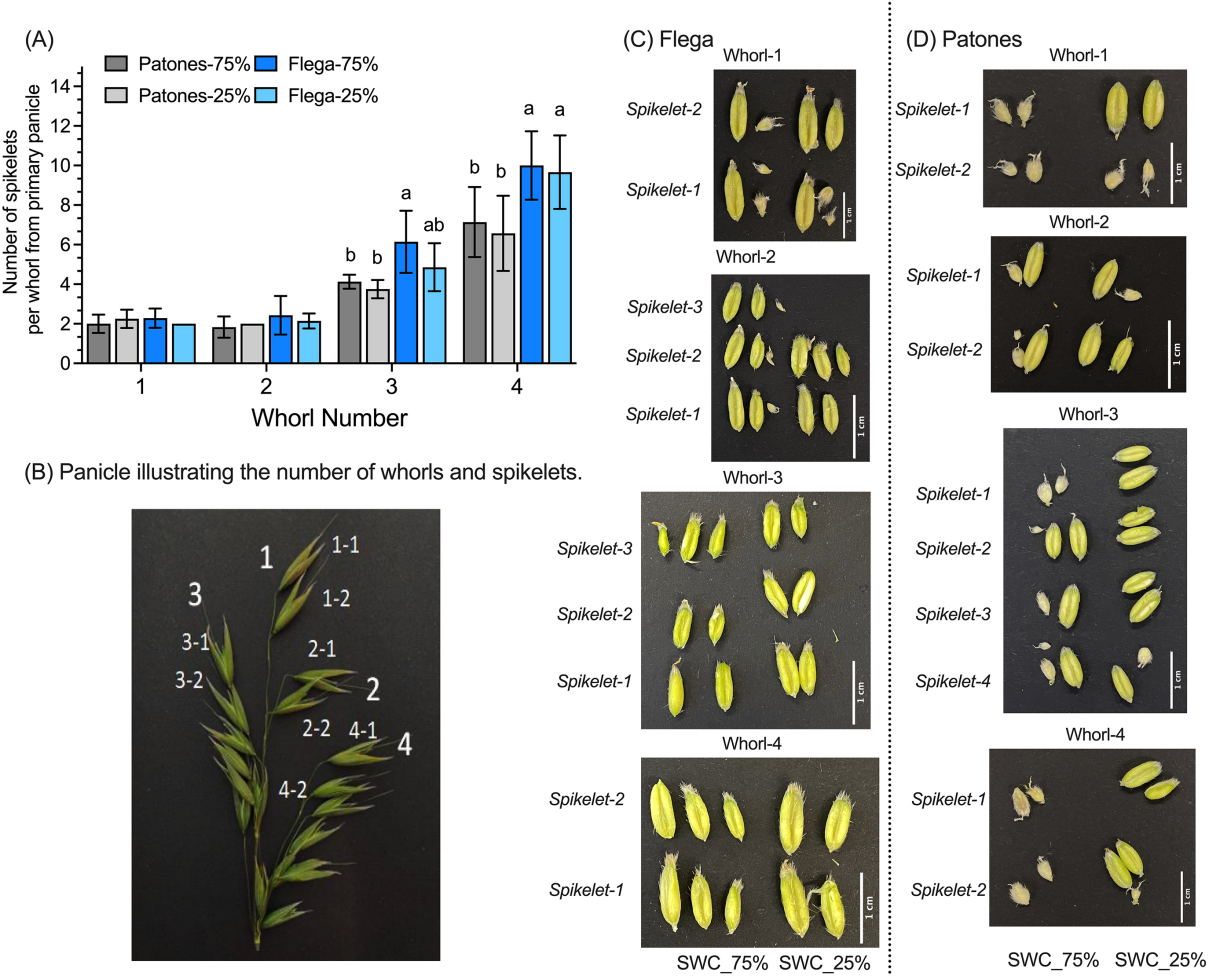
(A) Number of spikelets in each whorl in the primary panicle of Flega and Patones under SWC_75% (control) and SWC_25% (drought). The data shown are average ± SD (n = 8), with significant differences using ANOVA followed by Tukey's HSD test (*p* < *0.05*), indicated by lowercase letters. (B) A primary oat panicle illustrating the numbering of whorls numbered from top (1) to bottom (4). Each whorl has spikelets, which are also numbered from top to bottom. Top whorls 1 and 2 have less spikelets compared to bottom whorls 3 and 4. Top whorls 1 and 2 as well as bottom whorls 3 and 4, the first two spikelets are shown. The developing grains were dissected from the corresponding spikelets from the primary panicle of control and drought‐stressed plants of (C) Flega (left) and (D) Patones (right).

The number of fertile spikelets and phenotypes of developing grains from the top and bottom spikelets in each whorl of the primary panicle and the grains were visually analysed for any developmental anomalies in response to drought (Figure 4). The spikelet number in each whorl of the primary panicle was significantly (*p* < *0.05*) higher in the bottom than in the top whorls in both cultivars under control and drought conditions (Figure 4A). Prior to sampling, the whorls and spikelets in the primary panicle were separately classified (Figure 4B) to establish grain sizes in different whorls (Figure 4C, D). Flega showed no observable differences in the sizes of developing grains between control and drought (Figure 4C). However, developing grains in Patones, particularly from the bottom (basal) whorl (number 4), showed significantly (*p* < *0.05*) larger grains with drought compared to controls, suggesting accelerated grain development (Figure 4D).

To further elucidate how these could reflect metabolite changes within the source and sink tissues, we profiled the metabolome of sheath, flag leaf, rachis and developing grains of primary panicle in the two oat cultivars.

### 3.3 Drought‐induced metabolite patterns in source (sheath, flag leaf and rachis) and sink (developing grains) differ between oat cultivars, Flega and Patones

Source tissues, including the flag leaf (FL), sheath (S) rachis (R), and sink tissue (developing grains) were assessed by nontargeted metabolite profiling. Whilst Principal Component Analysis (PCA) was first employed, partial least squared‐discriminant analyses (PLS‐DA) of the derived *m/z* features better indicated altered metabolite profiles between source and sink tissues (Figure S2). Further, source tissues showed distinct clustering between control and drought conditions in both cultivars. Based on the variation explained by component 1, the greater metabolomic changes were likely to be seen in flag leaves compared to other source tissues. Each cultivar had its own drought‐specific response; especially the metabolite profile of drought‐stressed Patones showed a clear separation of flag leaf and rachis (Figure S2).

**FIGURE 5:**
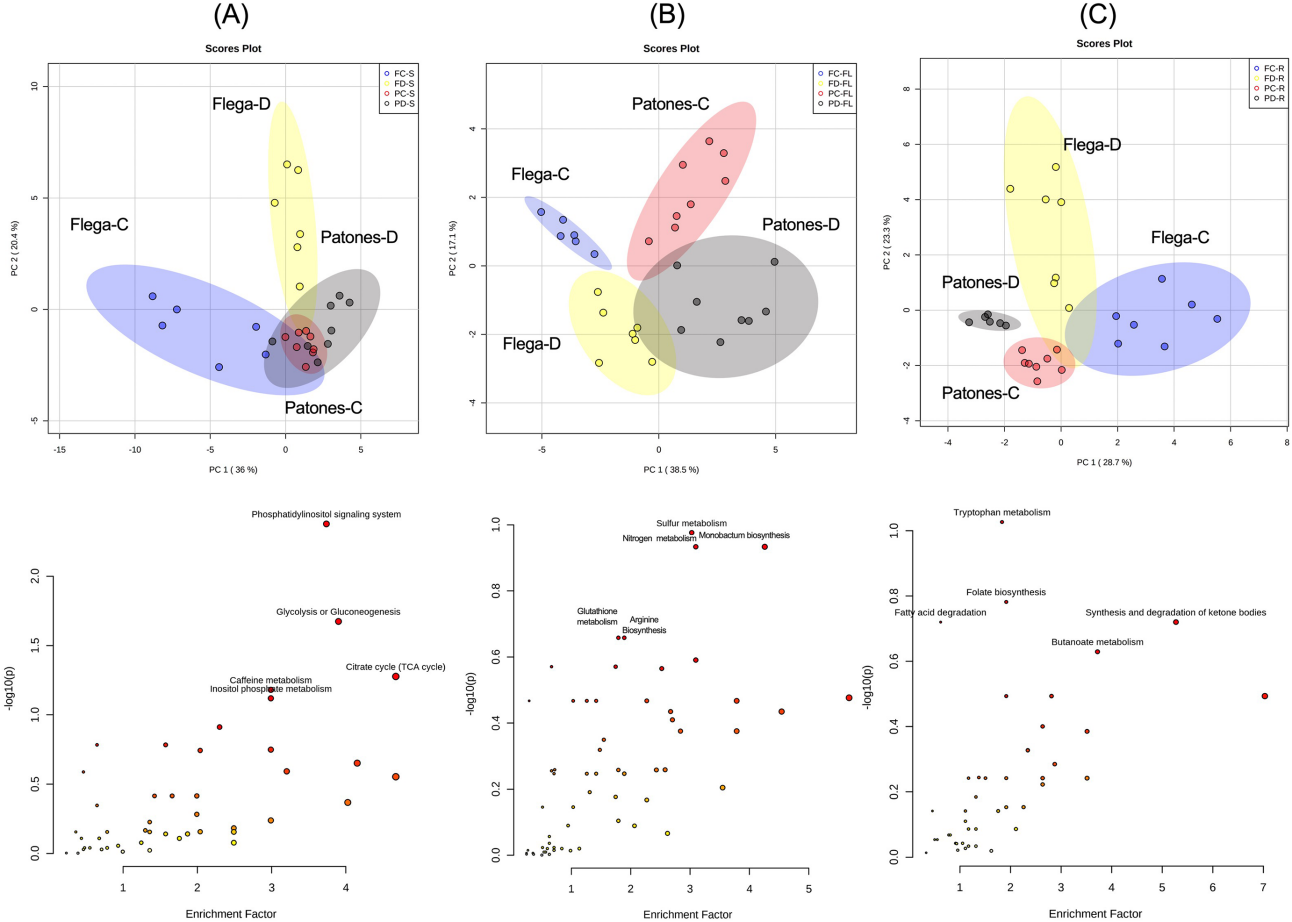
Principal component analysis (PCA) of annotated metabolites in different source tissues of Flega and Patones (A) Sheath‐S (B) Flag leaf‐FL and (C) Rachis‐R. Bottom panel shows the metabolite enrichment pathways associated with corresponding source tissues.

PCA analysis revealed a distinct shift in the sheath metabolite profiles in Flega under drought, whereas the control and drought groups showed greater similarities in Patones, indicating a less pronounced metabolite shift (Figure 5A). In the flag leaves, cultivar‐specific differences were prominent in the metabolome (across PC1), but drought‐responsive changes were seen in both cultivars across PC2 (Figure 5B). Distinctive metabolite responses were observed in the rachis with cultivars as well as different treatments (Figure 5C). The major metabolite sources of variation (as defined using ANOVA (parametric only) *post‐hoc* Fisher's LSD analysis [*p* < 0.05]) in the source tissues varied across sheath, flag leag and rachis. The enrichment of metabolites in sheath showed pathways related to phosphatidylinositol signaling system, glycolysis, the tricarboxylic acid (TCA) cycle and inositol phosphate metabolism. Flag leaf showed major metabolite pathways related to sulfur metabolism, nitrogen metabolism, glutathione biosynthesis and rachis showed tryptophan metabolism, fatty acid degradation, folate biosynthesis and synthesis and degradation of ketone bodies (Figure 5, **bottom panel**).

The initial metabolite assessments of developing grains showed major metabolome differences between the grains collected from top and bottom whorls. The sources of variation were identified and assessed by pathway enrichment analyses. Metabolite enrichment of grains from top whorls showed significant (*p* < *0.05*) metabolite hits associated with α‐linolenic acid metabolism, synthesis and degradation of ketone bodies. Conversely, pathways related to galactose metabolism, starch and sugar metabolism were enriched in the grains from bottom whorls (Figure S3).

Next, analyses focused on defining tissue‐specific metabolite profiles. Multiple comparisons indicated significant (*p* < 0.05) differences between metabolite accumulation patterns in Flega and Patones. One distinct difference in Patones was the accumulation of α‐linolenic acid in all source tissues under drought (Figure S4,A). In Flega, a key difference compared to Patones was the increase in the oxidative stress marker ophthalmic acid (OA) (Servillo et al., 2018) in flag leaves under drought (Figure S4,B). Genotypic‐ and treatment‐specific significant differences were also assessed in developing grains. In Patones, fatty acids, including α‐linolenic acid, were significantly reduced compared to Flega in the developing grains under drought (Figure S4,C). Given such differences, we next focused on defining key changes in each cultivar to elucidate distinctive metabolite‐wide responses to drought.

### 3.4 Drought‐related metabolite changes indicate an oxidative stress status in Flega

**FIGURE 6:**
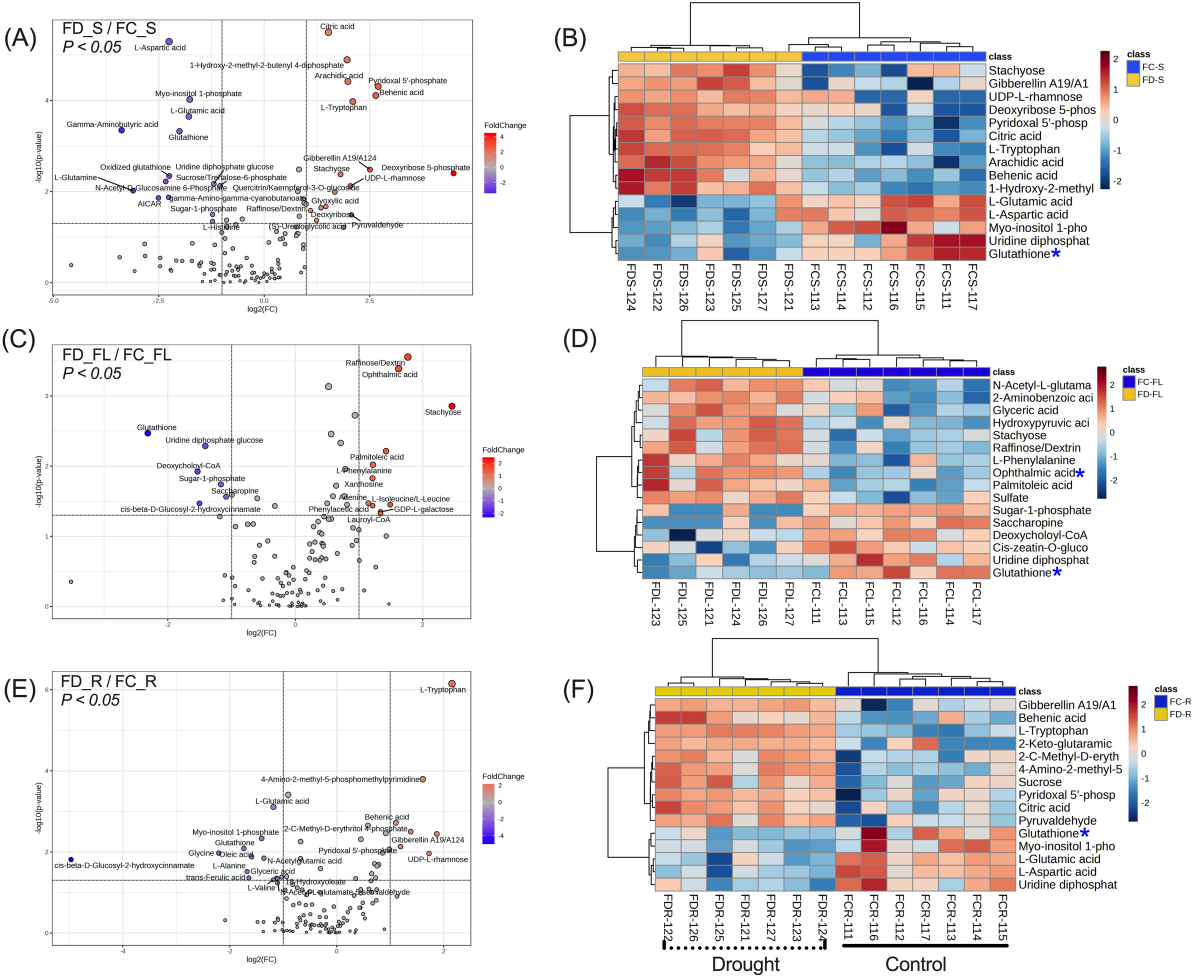
Volcano plot and Heat maps showing the levels of significant metabolites in (A, B) Sheath‐S, (C, D) Flag leaves‐FL, and (E, F) Rachis‐R of Flega control (FC) and drought (FD) plants. Blue asterisk ‘*’ indicates the levels of ophthalmic acid and glutathione in different source tissues.

A pair‐wise analysis based on metabolites from the source and sink tissues of Flega (F) grown under well‐watered and drought conditions was done to identify the key metabolites responding to drought treatment. Significantly up‐ and down‐regulated metabolites in different source tissues (> two‐fold change, *p* < 0.05) were characterized by volcano plots (Figure 6; Table S1). Key responses under drought included increases in sugars, raffinose family oligosaccharides (RFOs), organic acids, glutathione pool and TCA intermediates. In the sheath, drought resulted in higher levels of TCA and glycolysis intermediates (citric acid, pyridoxal 5′‐phosphate, deoxyribose‐5‐phosphate), sugars (raffinose, stachyose, rhamnose, deoxyribose), organic acids (glyoxylic acid, (S)‐Ureidoglycolic acid), pyruvaldehyde, gibberellin, behenic acid, arachidic acid, quercitrin/kaempferol‐3‐O‐glucoside and tryptophan. However, myo‐inositol‐1‐phosphate, sucrose‐6‐phosphate, uridine‐diphoshpate glucose, gamma‐aminobutyric acid (GABA), amino acids (glutamine, aspartic acid, glutamic acid, histidine), glutathione (GSH), oxidized glutathione (GSSG), and N‐acetyl‐D‐glucosamine phosphate were significantly lower (Figure 6A, B). In the flag leaf, there were elevated levels of stachyose (5.4‐fold), raffinose (3.4‐fold), amino acids (isoleucine, phenylalanine) and palmitoleic acid, lauroyl‐CoA. However, levels of saccharopine and glutathione were reduced (Figure 6C, D; Table S2). OA (Figure 6D indicated by a blue*) was found to be significantly (*p* < 0.05) up‐regulated (3.0‐fold) in the flag leaves under drought. The rachis showed a consistent increase of pyridoxal 5′‐phosphate, behenic acid, gibberellin, tryptophan, UDP‐L‐rhamnose, sucrose and citric acid with drought. Conversely, levels of amino acids (alanine, glycine, glutamic acid, valine), fatty acids (18‐hydroxyoleate, oleic acid), glyceric acid, N‐acetyl glutamic acid, myo‐inositol‐1‐phosphate, ferulic acid, and glutathione were lower in the rachis with drought (Figure 6E, F).

**FIGURE 7:**
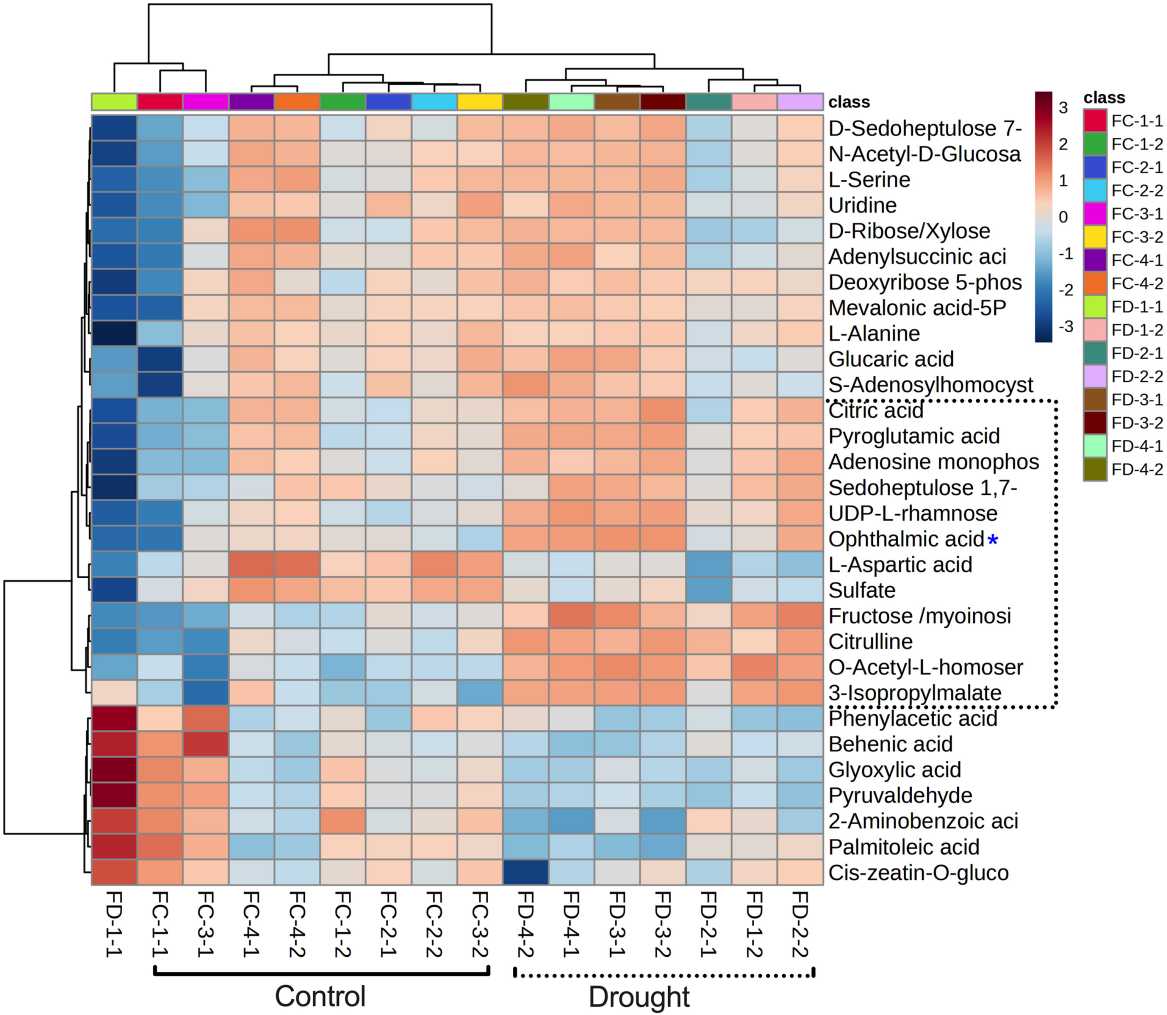
Heat map showing the metabolites accumulation pattern in the developing grains of Flega control and drought plants. Black dotted box indicates the key group of differential metabolites under drought and blue asterisk ‘*’ indicates the levels of ophthalmic acid in grains. Significant metabolites were identified based on p‐value threshold (*p* < *0.05*) using ANOVA (parametric only) followed by post‐hoc Fisher's LSD analysis. The heatmap was constructed using Euclidean methods for distance measure and Ward clustering. The metabolite levels were clustered by compounds (rows) and biological replicates (columns) per treatment. Eight individual plants were used as eight biological replicates.

Compared to well‐watered conditions, the developing grains from the apical (top whorl 1 and 2) and basal (bottom whorl 3 and 4) whorls in the primary tiller of Flega showed increased levels of citric acid, pyroglutamic acid, citrulline, 3‐isopropylmalate, O‐phosphoethanolamine, saccharopine and O‐acetyl‐L‐homoserine and lower levels of aspartic acid and sulfate (Figures 7, and S5). Interestingly, OA accumulation was most prominent in the apical grains of both control and droughted Flega plants. Overall, the younger grains collected from the basal spikelets showed an enhanced accumulation of TCA and glycolysis intermediates, sugars, sugar phosphates and amino acids under drought. These observations and the consistently lower levels of reduced glutathione (Figure 6 indicated by blue*) in the sheath, flag leaf and rachis aligned with the accumulation of ophthalmic acid to suggest increased oxidative stress status in Flega under drought.

### 3.5 Drought stress induces differential accumulation of fatty acids and metabolites related to lipid metabolism in Patones

**FIGURE 8:**
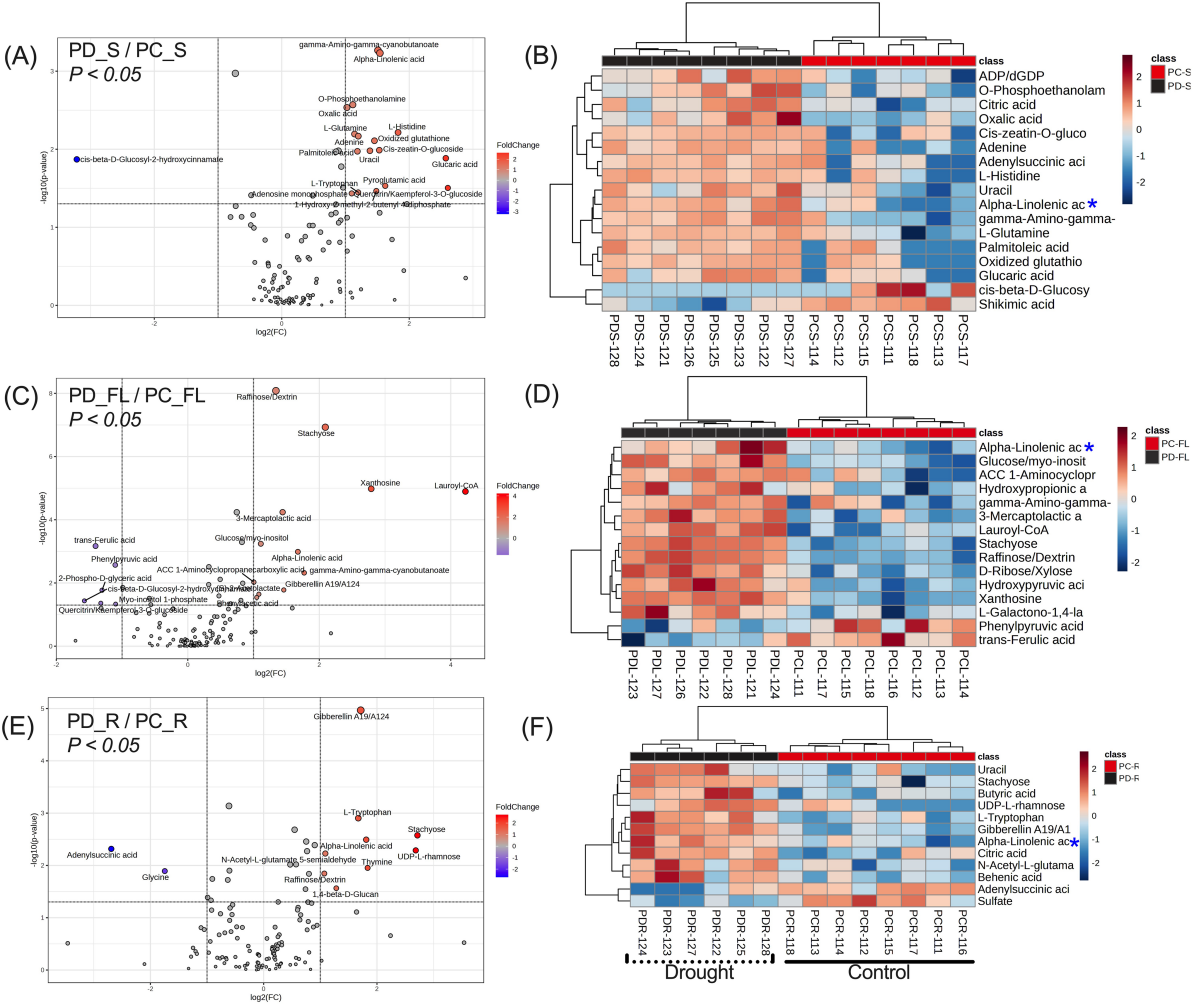
Volcano plot and Heat maps showing the levels of significant metabolites in (A, B) Sheath‐S, (C, D) Flag leaves‐FL, and (E, F) Rachis‐R of Patones control (FC) and drought (FD) plants. Blue asterisk ‘*’ indicates the levels of alpha‐linolenic acid in different source tissues.

Drought‐induced differential metabolite responses in all tissues of Patones were associated with lipid metabolism and fatty acids. The sheath showed a significant up‐regulation in TCA/glycolysis intermediates, amino acids and the organic acids citric acid, oxalic acid, glucaric acid, pyruvic acid as well as histidine, tryptophan, glutamine, oxidized glutathione and quercitrin (Figure 8A, B). Flag leaf from droughted plants showed up‐regulation in sugars such as raffinose, stachyose, ribose as well as the nucleoside xanthosine (Figure 8C, D). The rachis also showed increased levels of raffinose, stachyose, rhamnose, tryptophan and gibberellin (Figure 8E, F). Compared to controls, an 18‐fold increase in lauroyl‐CoA, which is involved in fatty acid elongation and degradation pathways, was observed in the flag leaf. Patones showed significantly increased accumulation of α‐linolenic acid in all three source tissues: sheath by 2.9‐fold, flag leaf by 3.1‐fold and in rachis by about 3.5‐fold (Figure 8; Table S2). This α‐linolenic acid accumulation pattern was not observed in Flega. The sheath also exhibited a drought‐induced decrease in glutathione levels, which was not seen in the flag leaf and rachis. As there were also no significant changes in OA, oxidative stress may not be as severe in Patones as in Flega.

**FIGURE 9:**
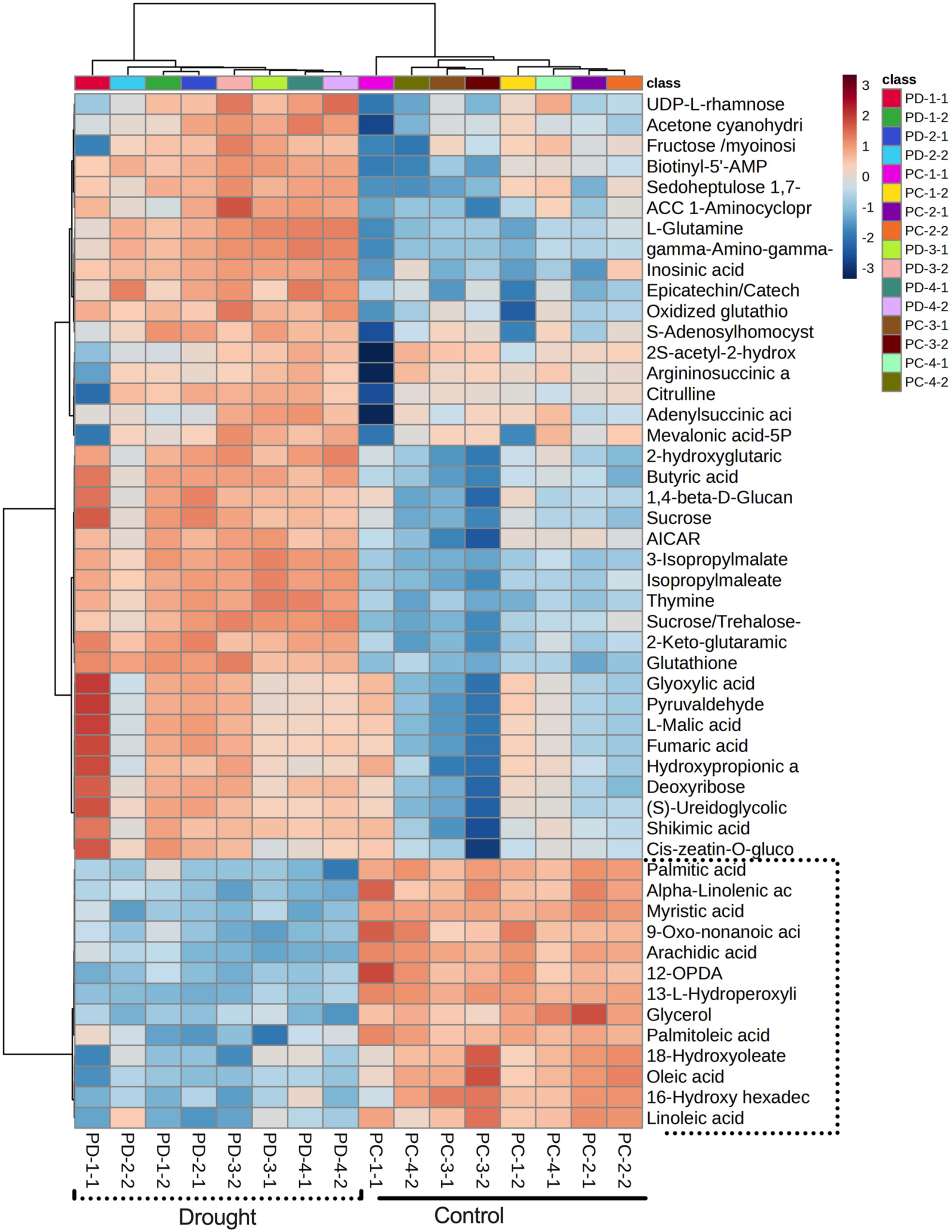
Heat map showing the normalized top 50 metabolite accumulation patterns in the developing grains from control and drought groups in Patones. Black dotted box indicates the key group of differential metabolites under drought. Significant metabolites of p‐value threshold (*p* < *0.05*) by ANOVA (parametric only) post‐hoc Fisher's LSD analysis are shown. The heatmap was generated using the Euclidean methods for distance measure and Ward clustering. The metabolite levels were clustered by compounds (rows) and biological replicates (columns) per treatment. Metabolite abundance is based on eight independent plants as biological replicates.

Compared to control, developing grains from droughted plants of Patones showed significant changes in fatty acids, sugars and amino acids. With drought, the developing grains showed decreased levels of fatty acid‐related metabolites (linoleic acid, oleic acid, palmitic acid, palmitoleic acid, myristic acid, linolenic acid, arachidic acid) (Figure 9). Levels of 12‐oxo‐phytodienoic acid (12‐OPDA) were also lower, which is a potentially important observation as it is a component of jasmonate biosynthesis and also a signal molecule that induces the expression of stress‐associated genes (Dave & Graham, 2012; Savchenko & Dehesh, 2014). Endogenous levels of reduced (GSH) and oxidized glutathione (GSSG) were significantly higher in grains from both upper and lower whorls under drought stress. Compared to grains from the top whorls, younger grains collected from the basal spikelets showed more significant metabolite changes under drought than the older grains. Younger grains showed increases in sugars, TCA and glycolysis intermediates, organic acids, and amino acids levels (Figure 9). These changes could be associated with accelerated and efficient grain‐filling, resulting in a larger grain size and early grain development in Patones under drought.

## 4 DISCUSSION

### 4.1 Drought imposed during anthesis suggests two different drought mechanisms in oats

The Mediterranean oat cultivars Flega and Patones exhibited differential morphological, physiological and metabolic responses to drought, which could represent important adaptive mechanisms. According to previous drought studies, the susceptible cultivar Flega tends to adopt a ‘water saving’ strategy, whereas the tolerant cultivar Patones exhibits a ‘water spending’ strategy (Canales et al., 2021). This is confirmed in this current study as Flega showed less water usage than Patones with drought (Figures 1 and 2). Morphologically, Flega exhibited faster flowering and reduced shoot biomass than Patones (Figure 3). However, Patones had greater evidence of accelerated grain development under anthesis‐applied drought (Figure 4). Given such distinctive drought responses by these Mediterranean oat cultivars, and the accelerated grain development phenotype, we employed metabolomic responses to characterise how this could reflect shifts in source‐sink relationships.

Source‐sink dynamics are complex and determined by multiple factors (Fabre et al., 2020); for example, biomass, relative CO_2_ levels and photosynthetic rates (Makino & Mae, 1999). Metabolically, the relative accumulation of photosynthetic end‐products (carbohydrates) in source tissues can reflect reduced sink demand and photosynthetic rates. This will also result in a systemic decrease in nitrogen content (such as amino acids) in the leaves (Zhang et al., 2022).

Considering source tissues, Flega showed increased raffinose, stachyose, citric acid, behenic acid, 3‐mercaptolactic acid, hydroxy pyruvic acid, and pyruvic acid levels but a lesser accumulation of many amino acids (e.g. glutamine, histidine, glutamic acid, aspartic acid except tryptophan) with drought (Figure S5). This could reflect the increased sink tissues demand (developing grains) as N influences the rate of grain‐filling and grain weight (Wei et al., 2019). Further, drought‐induced increase in bioenergy‐associated TCA intermediates, such as citric acid in the sheath, could be associated with increased mobilisation of sucrose (Figure 6B). The droughted flag leaves of Flega showed increased accumulation of oxidative stress marker, OA, along with TCA and glycolysis intermediates (Figure 6C, D). The similar trend in the accumulation of TCA intermediates and organic acids was also seen in rachis under drought with a consistent decrease in reduced glutathione levels, suggesting an increased cellular redox status in Flega. Such changes were not prominent in Patones, which instead showed increased accumulation of α‐linolenic acid in sheath, flag leaf and rachis under drought (Figure S4) along with a decreased accumulation of 12‐OPDA in grains. This suggests the possible regulatory role of jasmonates in this cultivar. Interestingly, there were increases in the raffinose family of oligosaccharides (RFOs) in the source tissues of both cultivars. RFOs are a‐1, 6‐galactosyl extensions of sucrose that accumulate in seeds by phloem loading/transport and serve as desiccation protectants (Ma et al., 2021). Taken together, our results suggested some overlapping responses but also distinctive metabotypes in the two oat cultivars under drought.

### 4.2 Accelerated grain‐filling under drought stress may be mediated by jasmonates in Patones

Lipids are essential components in maintaining the structural integrity of cells and organelles, but lipid derivatives also have roles in signalling events inducing the defences against abiotic stimuli (Sharma et al., 2023) and regulating cell metabolism (Kim, 2020). In plants, the most predominant unsaturated fatty acids (UFAs) are 18‐carbon (C18), including oleate, linoleate, and α‐linoleate, which can act as antioxidants and also feed into the production of jasmonates (Seth et al., 2024). Jasmonates include jasmonic acid (JA) and its intermediate 12‐OPDA (Savchenko et al., 2014). Numerous studies have shown the role of JA in regulating abiotic and biotic stress responses. In this study, we have observed changes in linolenic acid and the accumulation of JA‐associated metabolites that could be related to drought‐induced accelerated grain development in oat. Earlier studies in Patones and Flega by Sánchez‐Martín et al. (2018) found that drought elicited linolenic acid biosynthesis, leading to the accumulation of JAs in Patones under drought stress. Similarly, we observed a consistent increase in α‐linolenic acid in the vegetative tissues of Patones, which were not prominent in Flega (Figures 8 **and** S4). Interestingly, 12‐OPDA content in all three source tissues of Patones, (PD‐S, PD‐FL, PD‐R) did not show significant change but decreased in grains under drought. Reduced levels of 12‐OPDA during drought could suggest increased flux towards jasmonic acid biosynthesis (Figure 9). Given the results, fatty acids, specifically α‐linolenic acid, may be triggering JA events to influence sink‐to‐source relationships (Dave & Graham, 2012; Zi et al., 2022) in Patones under drought.

Such signalling is likely to contribute to grain development and yield (Huang et al., 2017; Sohn et al., 2022). Transgenic rice plants overexpressing the Arabidopsis JA carboxyl methyltransferase gene (*AtJMT*) with high levels of methyl jasmonate (MeJA) showed a significant reduction in grain yield when exposed to drought stress (Kim et al., 2009). More compelling, the wheat *triticale grain weight 1* (*tgw1*) mutant with reduced grain weight has been shown to encode *KETO‐ACYL THIOLASE 2B* (KAT‐2B), which is involved in a peroxisomal step in JA biosynthesis (Chen et al., 2020). Such studies suggest that jasmonates could be key in driving accelerated grain development. This being stated, the *tgw1* mutation also compromised the expression of the gibberellin biosynthesis gene *ENT‐KAURENE SYNTHASE*. In this context, it should be noted that we observed a significant accumulation of gibberellin in Patones subjected to drought (Figure 8). Therefore, it is likely that the accelerated grain‐filling phenotype reflects the interplay of JA with other hormones. Further work is crucial to dissect how such hormone interactions could influence grain development and yield during drought stress in oats.

### 4.3 Drought‐induced oxidative stress in Flega could induce early flowering

Compared to Patones, the drought‐sensitive cultivar Flega showed an early flowering response both in control and drought conditions. Drought tolerance mechanism in plants can promote flowering by shortening their life cycle via earlier up‐regulation of flowering genes such as *GI (GIGANTEA), TSF (TWIN SISTER OF FT), FT (FLOWERING LOCUS T) and SOC1 (SUPPRESSOR OF OVEREXPRESSION OF CONSTANS 1)* (Liang et al., 2019). A common observation in Flega was a shift in reduced GSH/oxidized GSSG status as well as OA accumulation, which most likely reflected oxidative stress and a shift in cellular redox potential during drought. OA is synthesised from glutamate and 2‐ABA in oat, barley, wheat, and rye (Servillo et al., 2018); increased OA is paralleled by a decrease in GSH and an increase in isoleucine biosynthesis. The generation of reactive oxygen species (ROS) can degrade lipids, proteins and carbohydrates, shifting the cell's oxidative status (Kutasy et al., 2021). Grains of Flega in control and drought‐treated plants exhibited similar metabolite profiles, although water deficit conditions resulted in increased accumulation of myo‐inositol and TCA/glycolysis intermediates (Figure 7). This might suggest a change in energy status and could be associated with oxidative stress and ROS accumulation. Oxidative stress can accelerate stress‐induced early flowering, and this has been extensively characterised in Arabidopsis. This model species was used to show how *OXIDATIVE STRESS 2 (OXS2)* activates an early‐flowering stress escape response by indirectly interacting with florigen *FT* and transcription factor *FD* (*FLOWERING LOCUS D*) (Blanvillain et al., 2011; Liang & Ow, 2019). *OXIDATIVE STRESS 3* (*OXS3*) can interact with *SOC1* (*SUPPRESSOR OF OVEREXPRESSION OF CONSTANS 1*) and is involved in stress‐induced flowering through *AP1* (*APETALA 1*) (Liang et al., 2019). Equally, the signalling molecule myo‐inositol phosphatase (MIPS) (Du et al., 2011) could contribute to accelerated flowering in Flega. MIPS is associated with numerous stress responses and phosphoinositide (PI) signalling (Sharma et al., 2020). This could be an important observation that requires separate consideration in future studies.

## 5 CONCLUSION

**FIGURE 10:**
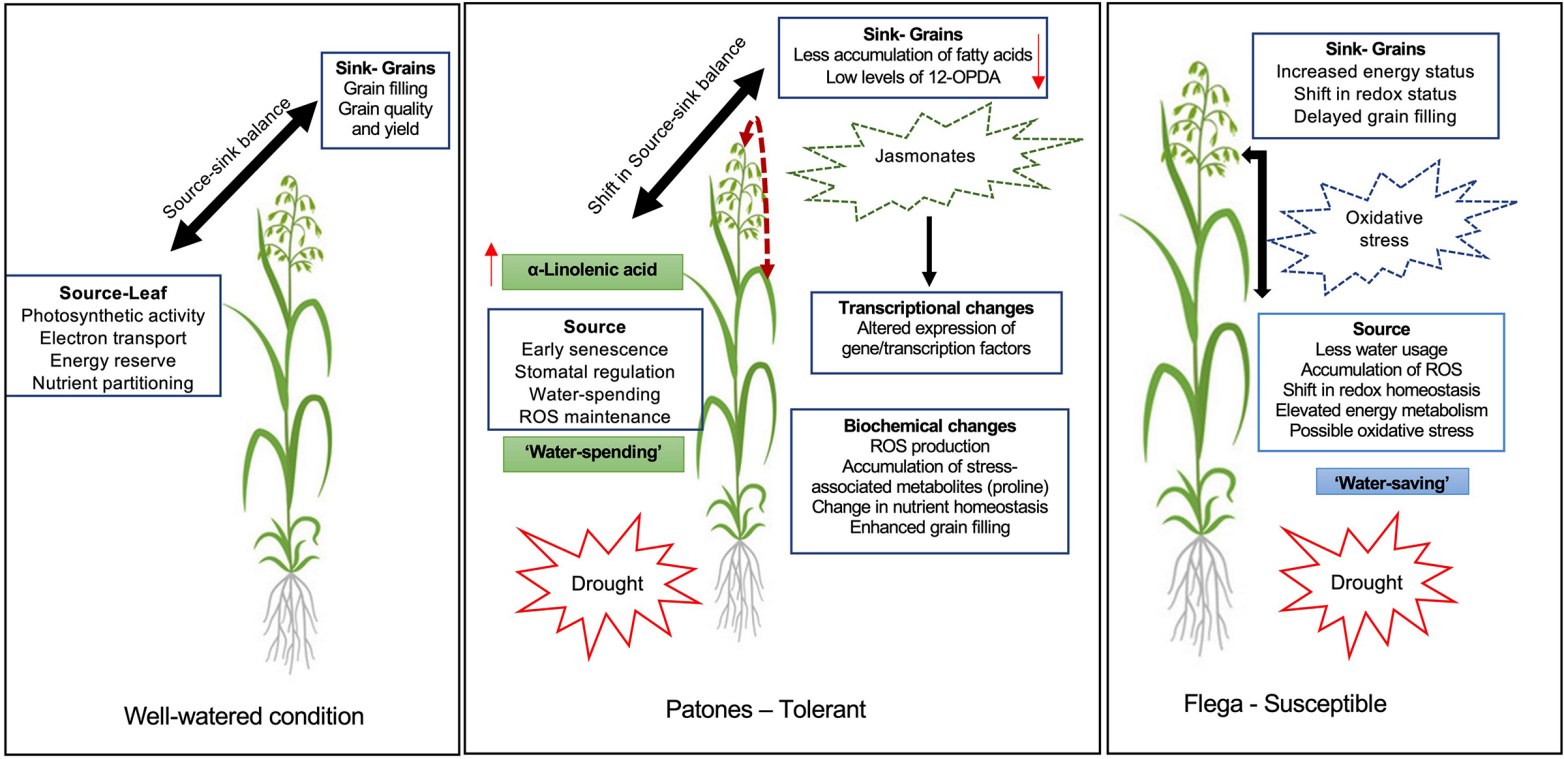
Schematic hypothetical representation of drought mechanism in Patones and Flega under drought.

This is the first report providing a comparative metabolomic approach identifying drought‐induced responses in two oat cultivars (Flega and Patones) with contrasting drought tolerance. Two differential biochemical mechanisms associated with accelerated grain development in response to drought were observed and we present the putative mechanisms in Figure 10.

Each cultivar showed distinct and significant metabolite differences under drought. However, the metabolic response to drought stress is variable depending on the tissues, developmental stages and on genetic background. Patones showed differential accumulation of C18 FA's and JA derivatives in source organs and grains under drought, which may be linked to JA‐induced signalling drought responses. However, in Flega, we observed a shift in the central energy metabolism along with an accumulation of oxidative stress markers that may be linked to an accelerated flowering phenotype to escape drought. Further dissection of these mechanisms with transcriptome studies could provide fundamental information regarding the central regulators that control accelerated grain development and their role in maintaining the source and sink relationships with potential application in improving oat yield and grain quality under drought‐prone environments.

## AUTHOR CONTRIBUTIONS

AG, LM and JD designed the study. AG and FC conducted and monitored the experiments at NPPC; FC, JB and KW imaging, data extraction and analysis from Lemnatec phenomics platform in NPPC. AG, FJC, BH and RD conducted morphological, physiological measurements, grain imaging, metabolite sampling and extraction. HP contributed to metabolite run, MB and AG performed metabolite data analysis. FJC contributed to morphological and physiological and statistical analysis for morphological and physiological traits. AG, FJC, EP and LM interpreted the results and drafted the initial manuscript. AG, BH, EP, FC, FJC, JD, KW and LM contributed to final preparation of manuscript and all authors reviewed the manuscript.

## FUNDING INFORMATION

AG, JD and LM acknowledge support from the Biotechnology and Biological Sciences Research Council (UK) Grains for Health grant (BBS/E/W/0012843A) and the Healthy‐Oats (Wales‐Ireland Interregional fund). AG is further supported by Strategic Program for Resilient Crops: Grains for Health (BBSRC grant, BBS/E/IB/230001B). The National Plant Phenomic Platform acknowledges Horizon2020 support via project EPPN2020 Grant agreement ID: 731013. EP acknowledge support from grant [PID2022‐142574OB‐I00] funded by MICIU/AEI /10.13039/501100011033, FEDER, UE, and Junta de Andalucia [QUAL21_023 IAS]. FC was supported by a ‘Margarita Salas’ post‐doctoral fellowship (UCO01MS) from the University of Cordoba (Requalification of the Spanish university system) from the Ministry of Universities financed by the European Union (NexGenerationEU).

## CONFLICT OF INTEREST STATEMENT

The authors declare that the research was conducted in the absence of any commercial or financial relationships that could be construed as a potential conflict of interest.

## Supporting information


**Data S1**.

**Data S2**.

## Data Availability

Data supporting the findings in this study are available within the paper and supplementary data published online. Nontargeted metabolomic data can be found in Tables S1 and S2.
