# Supplementary material for: Metabolomic approaches suggest two mechanisms of drought response post‐anthesis in Mediterranean oat (Avena sativa L.) cultivars
Source: Physiol Plant. 2025 Mar 27;177(2):e70181. doi: 10.1111/ppl.70181 (PMC11949858; doi:10.1111/ppl.70181)
Supplement: Supplementary file 1 — Data S1. [file PPL-177-e70181-s001.pdf]

Metabolomic approaches suggest two mechanisms of drought response post-anthesis in Mediterranean oat (Avena sativa L.) cultivars

Aiswarya Girija[1,2], Francisco J Canales[1,3], Bahareh Sadat Haddadi[1], Rachel Dye[2], Fiona Corke[4], Kevin Williams[4], Helen Phillips[1], Manfred Beckmann[1], Elena Prats[5], John H Doonan[4], Luis A J Mur[1]

**Supplementary Figures**

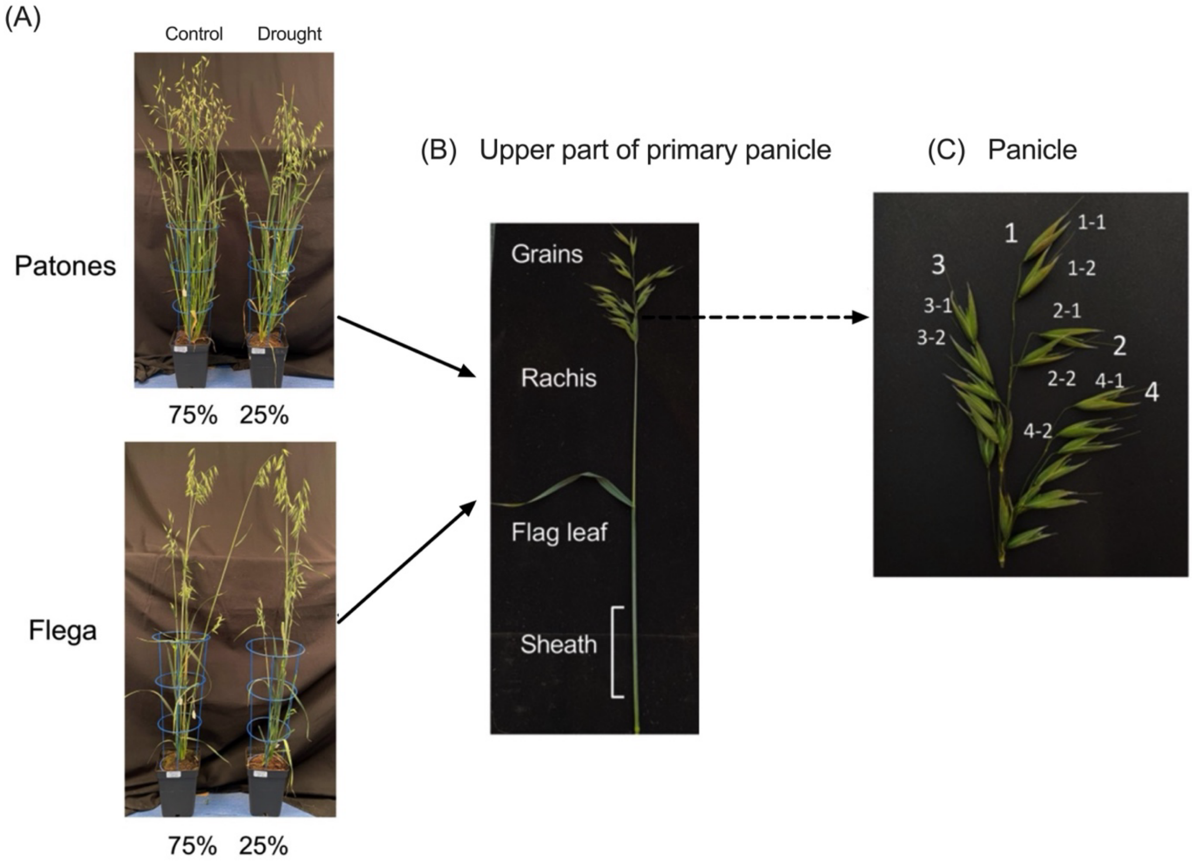


**Figure S1: (A)** Patones and Flega at 75% SWC (control-left) and 25% SWC (drought-right). **(B)** The primary panicle was harvested at growth stage (GS)75. **(C)** The primary panicle inflorescence is divided into separate whorls. Spikelet differentiation occurs at the tip (“1”) and proceeds basally (sequentially “2”,”3” and “4”), and within spikelet floret develop acropetally. The top two spikelets from apical or top (1,2) and basal or bottom whorls (3,4) were harvested. The experiment was conducted on a Lematec platform at the National Plant Phenomics Centre (NPPC) under controlled conditions

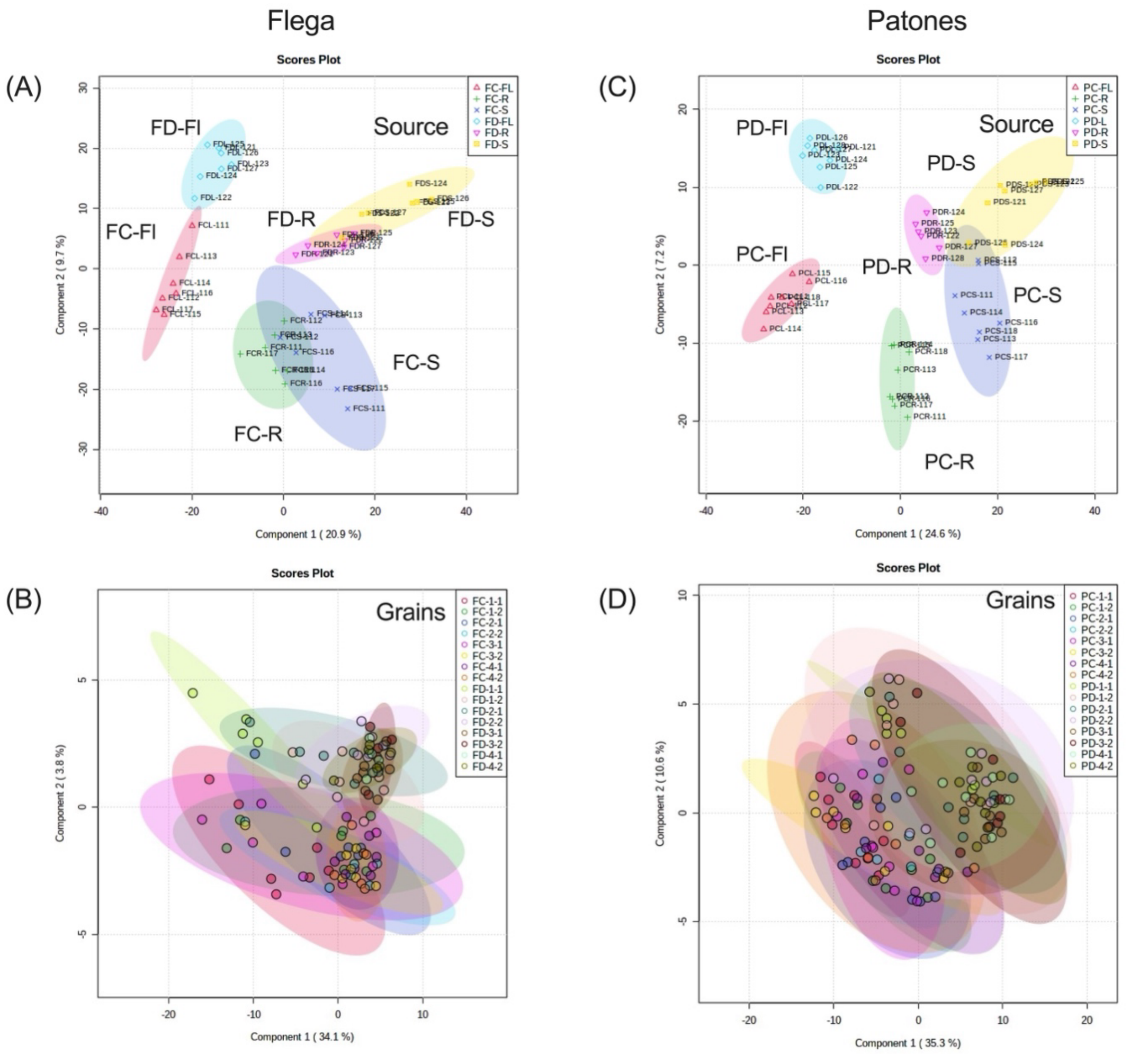


**Figure S2:** PCA showing metabolite variation of all m/z features captured in negative mode (A) Top panel shows metabolite distribution **(A)** in Flega - source tissues and **(B)** in Flega - grains **(C)** Patones -source and **(D)** Patones - grains under well-watered and drought conditions.

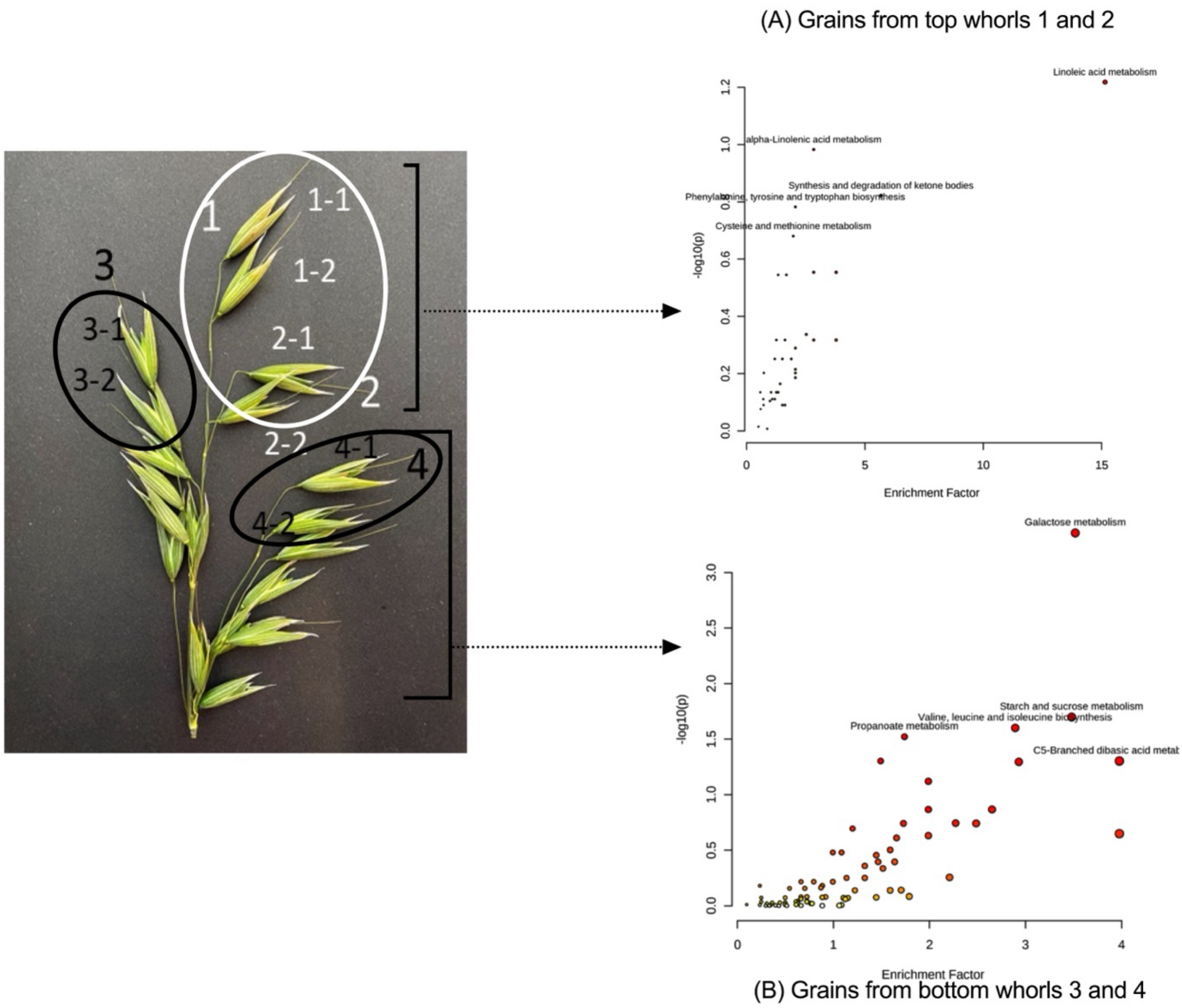


**Figure S3:** Metabolite enrichment pathways in developing grains of Flega and Patones collected from **(A)** top whorls 1 and 2 and **(B)** bottom whorls 3 and 4.

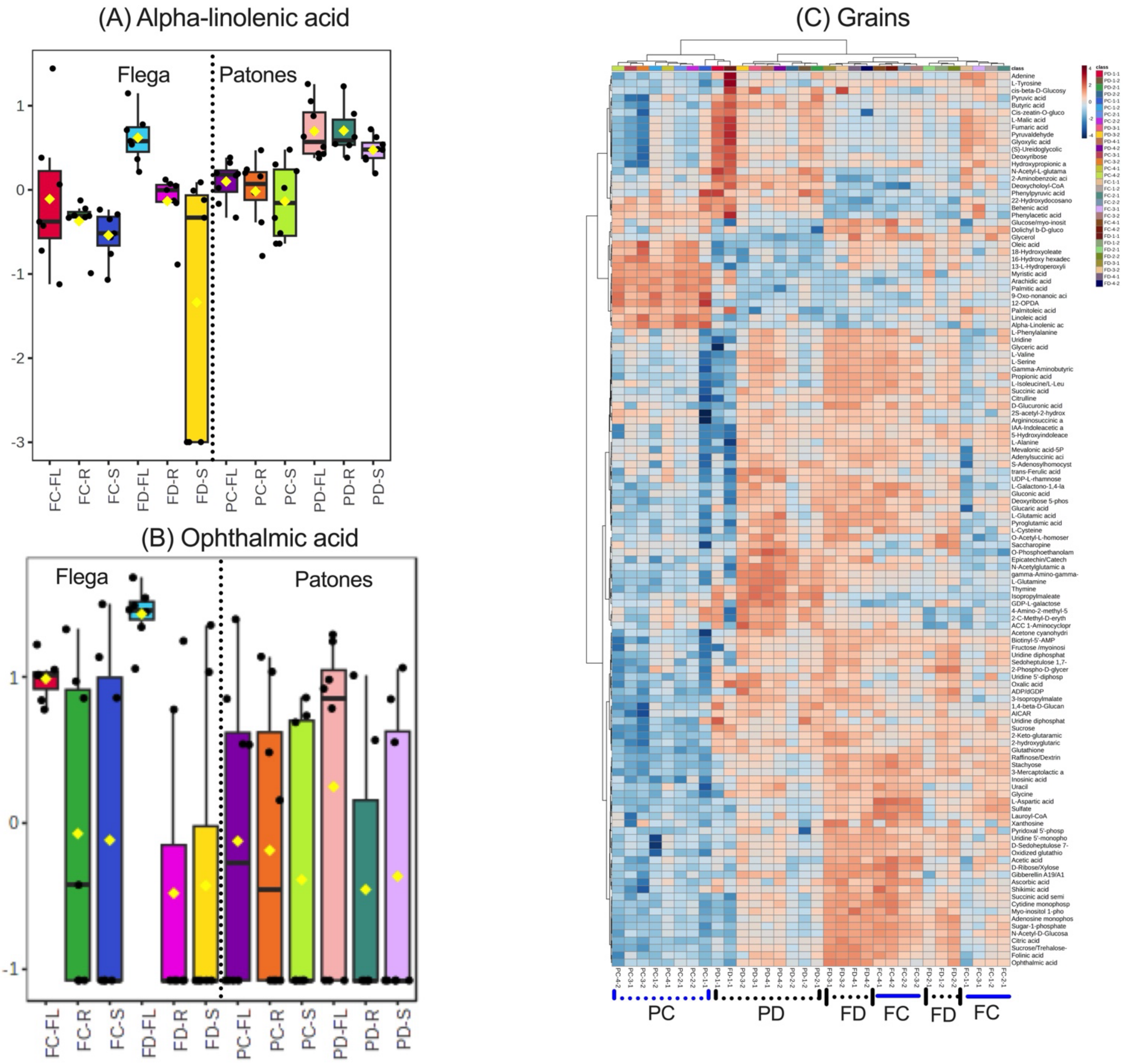


**Figure S4:** Box plot showing the accumulation of **(A)** alpha-linolenic acid and **(B)** ophthalmic acid in source tissues of Flega and Patone under control-C and drought-D. **(C)** Heat map showing metabolite accumulation pattern in developing grains of Flega and Patones under well-watered and drought conditions. Top significant metabolites of p-value threshold (*P* < *0.05*) by ANOVA (parametric only) post-hoc Fisher's LSD analysis is shown.

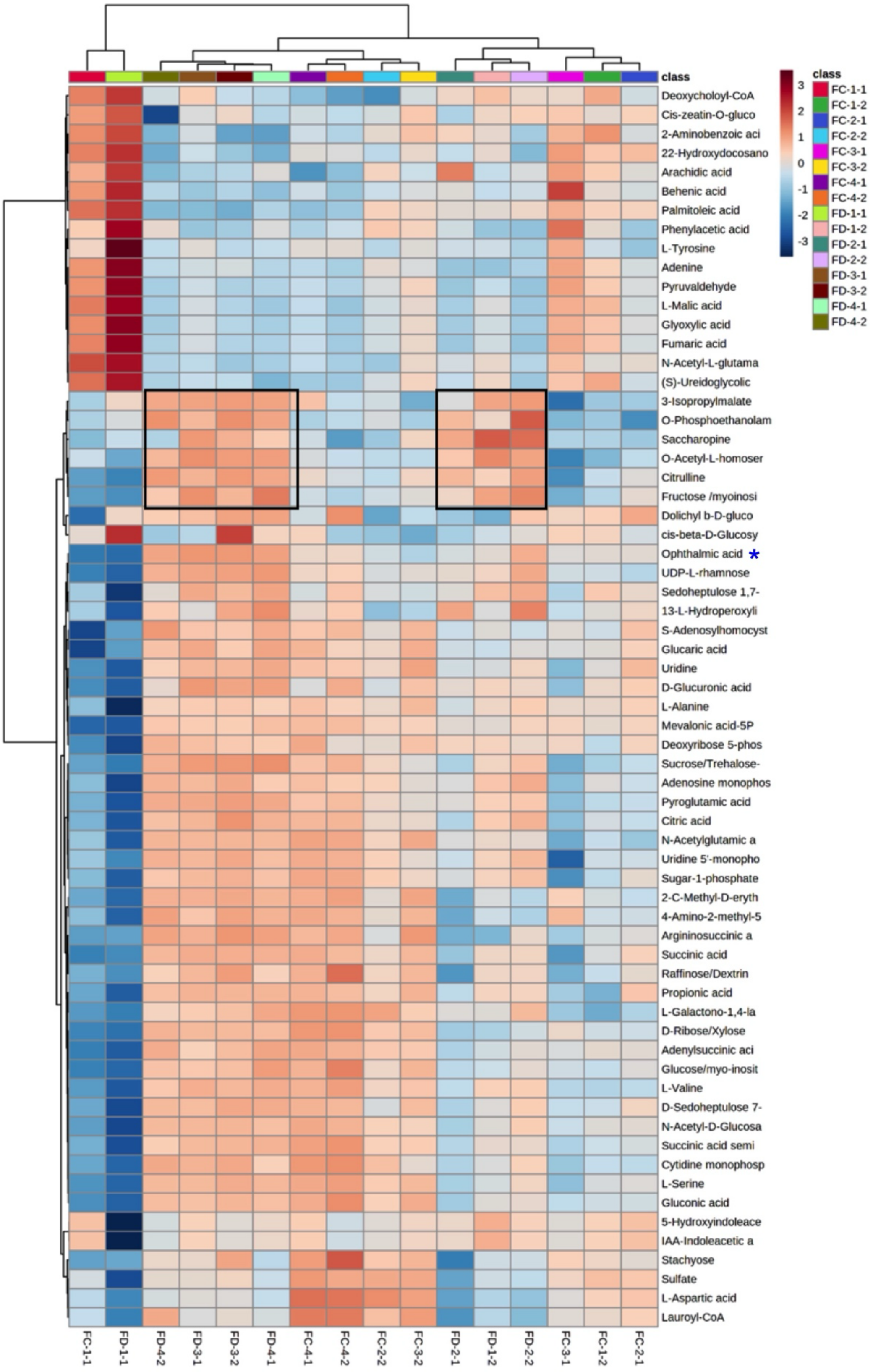


**Figure S5:** Heat map showing metabolite accumulation pattern in developing grains of Flega under well-watered and drought conditions. Top significant metabolites of p-value threshold ($P < 0.05$) by ANOVA (parametric only) post-hoc Fisher's LSD analysis is shown.
